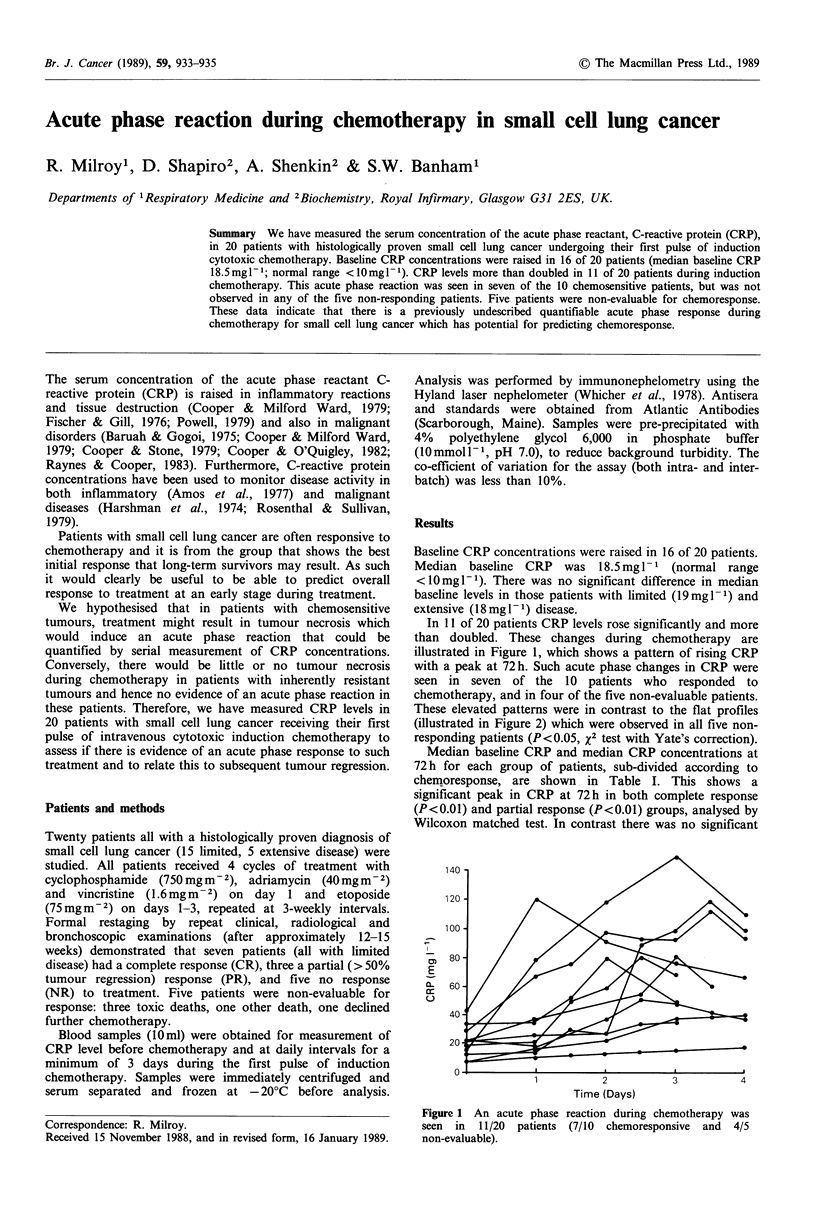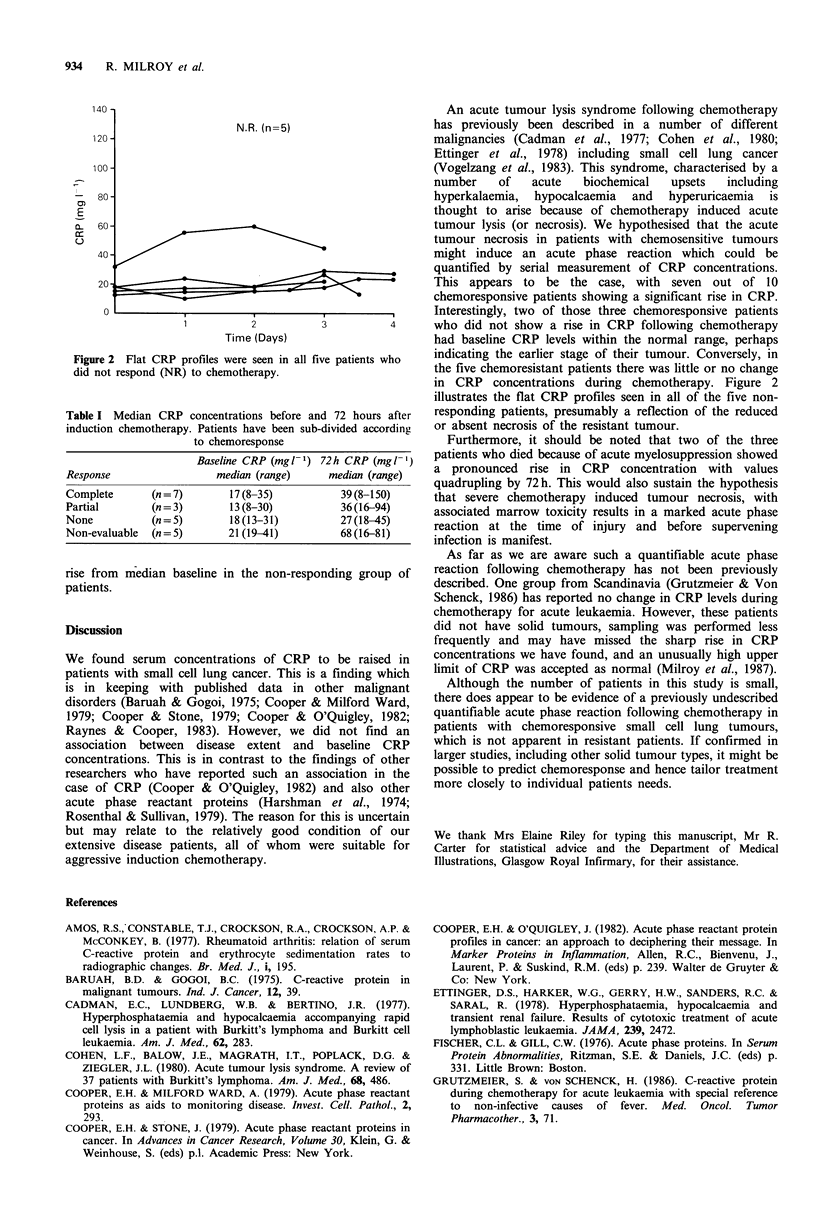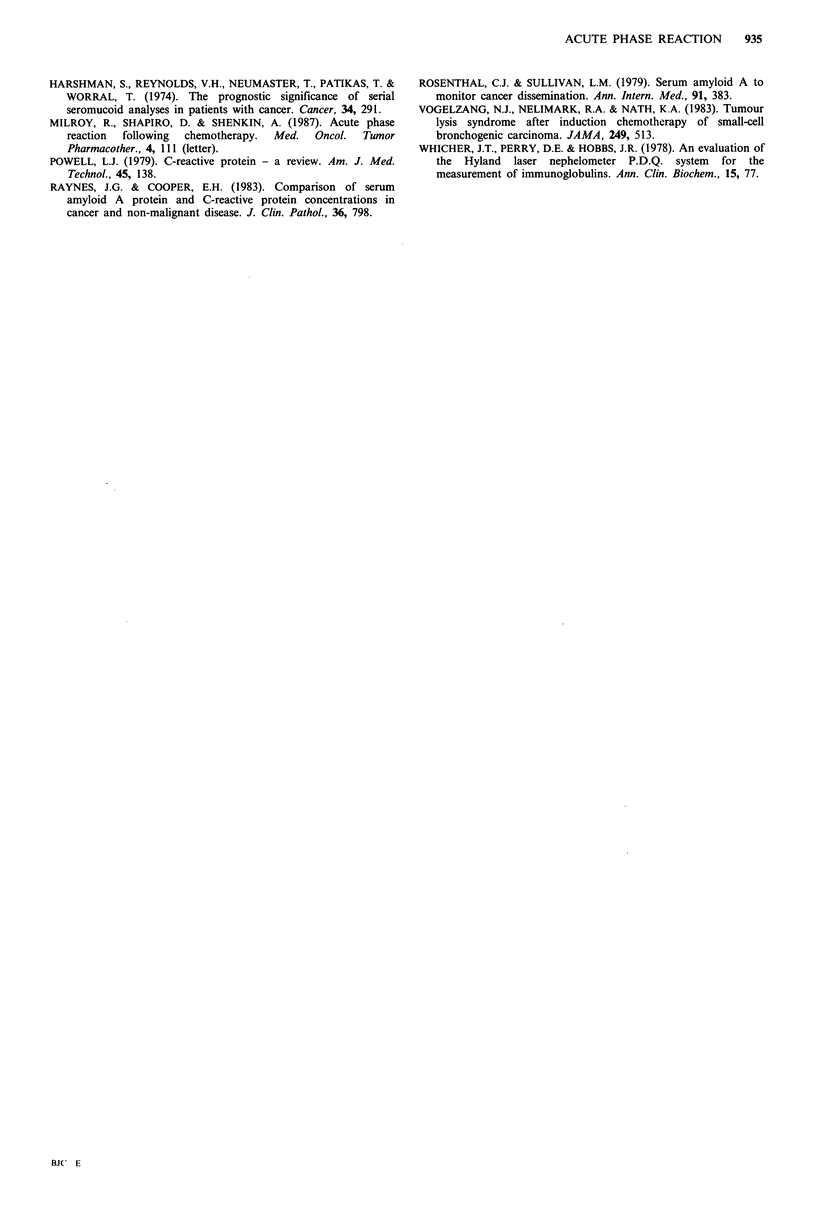# Acute phase reaction during chemotherapy in small cell lung cancer.

**DOI:** 10.1038/bjc.1989.197

**Published:** 1989-06

**Authors:** R. Milroy, D. Shapiro, A. Shenkin, S. W. Banham

**Affiliations:** Department of Respiratory Medicine, Royal Infirmary, Glasgow, UK.

## Abstract

We have measured the serum concentration of the acute phase reactant, C-reactive protein (CRP), in 20 patients with histologically proven small cell lung cancer undergoing their first pulse of induction cytotoxic chemotherapy. Baseline CRP concentrations were raised in 16 of 20 patients (median baseline CRP 18.5 mg l-1; normal range less than 10 mg l-1). CRP levels more than doubled in 11 of 20 patients during induction chemotherapy. This acute phase reaction was seen in seven of the 10 chemosensitive patients, but was not observed in any of the five non-responding patients. Five patients were non-evaluable for chemoresponse. These data indicate that there is a previously undescribed quantifiable acute phase response during chemotherapy for small cell lung cancer which has potential for predicting chemoresponse.


*Br. J. Cancer* (1989), **59**, 933–935 © The Macmillan Press Ltd., 1989

### Acute phase reaction during chemotherapy in small cell lung cancer

R. Milroy[1], D. Shapiro[2], A. Shenkin[2] & S.W. Banham[1]

*Departments of [1]Respiratory Medicine and [2]Biochemistry, Royal Infirmary, Glasgow G31 2ES, UK.*

**Summary** We have measured the serum concentration of the acute phase reactant, C-reactive protein (CRP), in 20 patients with histologically proven small cell lung cancer undergoing their first pulse of induction cytotoxic chemotherapy. Baseline CRP concentrations were raised in 16 of 20 patients (median baseline CRP $18.5\,mg\,l^{-1}$; normal range $<10\,mg\,l^{-1}$). CRP levels more than doubled in 11 of 20 patients during induction chemotherapy. This acute phase reaction was seen in seven of the 10 chemosensitive patients, but was not observed in any of the five non-responding patients. Five patients were non-evaluable for chemoresponse. These data indicate that there is a previously undescribed quantifiable acute phase response during chemotherapy for small cell lung cancer which has potential for predicting chemoresponse.

The serum concentration of the acute phase reactant C-reactive protein (CRP) is raised in inflammatory reactions and tissue destruction (Cooper & Milford Ward, 1979; Fischer & Gill, 1976; Powell, 1979) and also in malignant disorders (Baruah & Gogoi, 1975; Cooper & Milford Ward, 1979; Cooper & Stone, 1979; Cooper & O'Quigley, 1982; Raynes & Cooper, 1983). Furthermore, C-reactive protein concentrations have been used to monitor disease activity in both inflammatory (Amos *et al.*, 1977) and malignant diseases (Harshman *et al.*, 1974; Rosenthal & Sullivan, 1979).

Patients with small cell lung cancer are often responsive to chemotherapy and it is from the group that shows the best initial response that long-term survivors may result. As such it would clearly be useful to be able to predict overall response to treatment at an early stage during treatment.

We hypothesised that in patients with chemosensitive tumours, treatment might result in tumour necrosis which would induce an acute phase reaction that could be quantified by serial measurement of CRP concentrations. Conversely, there would be little or no tumour necrosis during chemotherapy in patients with inherently resistant tumours and hence no evidence of an acute phase reaction in these patients. Therefore, we have measured CRP levels in 20 patients with small cell lung cancer receiving their first pulse of intravenous cytotoxic induction chemotherapy to assess if there is evidence of an acute phase response to such treatment and to relate this to subsequent tumour regression.

### Patients and methods

Twenty patients all with a histologically proven diagnosis of small cell lung cancer (15 limited, 5 extensive disease) were studied. All patients received 4 cycles of treatment with cyclophosphamide ($750\,mg\,m^{-2}$), adriamycin ($40\,mg\,m^{-2}$) and vincristine ($1.6\,mg\,m^{-2}$) on day 1 and etoposide ($75\,mg\,m^{-2}$) on days 1–3, repeated at 3-weekly intervals. Formal restaging by repeat clinical, radiological and bronchoscopic examinations (after approximately 12–15 weeks) demonstrated that seven patients (all with limited disease) had a complete response (CR), three a partial (>50% tumour regression) response (PR), and five no response (NR) to treatment. Five patients were non-evaluable for response: three toxic deaths, one other death, one declined further chemotherapy.

Blood samples (10 ml) were obtained for measurement of CRP level before chemotherapy and at daily intervals for a minimum of 3 days during the first pulse of induction chemotherapy. Samples were immediately centrifuged and serum separated and frozen at −20°C before analysis.

Correspondence: R. Milroy.
Received 15 November 1988, and in revised form, 16 January 1989.

Analysis was performed by immunonephelometry using the Hyland laser nephelometer (Whicher *et al.*, 1978). Antisera and standards were obtained from Atlantic Antibodies (Scarborough, Maine). Samples were pre-precipitated with 4% polyethylene glycol 6,000 in phosphate buffer ($10\,mmol\,l^{-1}$, pH 7.0), to reduce background turbidity. The co-efficient of variation for the assay (both intra- and inter-batch) was less than 10%.

### Results

Baseline CRP concentrations were raised in 16 of 20 patients. Median baseline CRP was $18.5\,mg\,l^{-1}$ (normal range $<10\,mg\,l^{-1}$). There was no significant difference in median baseline levels in those patients with limited ($19\,mg\,l^{-1}$) and extensive ($18\,mg\,l^{-1}$) disease.

In 11 of 20 patients CRP levels rose significantly and more than doubled. These changes during chemotherapy are illustrated in Figure 1, which shows a pattern of rising CRP with a peak at 72 h. Such acute phase changes in CRP were seen in seven of the 10 patients who responded to chemotherapy, and in four of the five non-evaluable patients. These elevated patterns were in contrast to the flat profiles (illustrated in Figure 2) which were observed in all five non-responding patients ($P<0.05$, $\chi^2$ test with Yate's correction).

Median baseline CRP and median CRP concentrations at 72 h for each group of patients, sub-divided according to chemoresponse, are shown in Table I. This shows a significant peak in CRP at 72 h in both complete response ($P<0.01$) and partial response ($P<0.01$) groups, analysed by Wilcoxon matched test. In contrast there was no significant

**Figure 1** An acute phase reaction during chemotherapy was seen in 11/20 patients (7/10 chemoresponsive and 4/5 non-evaluable).

**Figure 2** Flat CRP profiles were seen in all five patients who did not respond (NR) to chemotherapy.

**Table I** Median CRP concentrations before and 72 hours after induction chemotherapy. Patients have been sub-divided according to chemoresponse

| *Response* | | *Baseline CRP* ($mg\,l^{-1}$) *median* (*range*) | *72h CRP* ($mg\,l^{-1}$) *median* (*range*) |
|---|---|---|---|
| Complete | ($n$=7) | 17 (8–35) | 39 (8–150) |
| Partial | ($n$=3) | 13 (8–30) | 36 (16–94) |
| None | ($n$=5) | 18 (13–31) | 27 (18–45) |
| Non-evaluable | ($n$=5) | 21 (19–41) | 68 (16–81) |

rise from median baseline in the non-responding group of patients.

### Discussion

We found serum concentrations of CRP to be raised in patients with small cell lung cancer. This is a finding which is in keeping with published data in other malignant disorders (Baruah & Gogoi, 1975; Cooper & Milford Ward, 1979; Cooper & Stone, 1979; Cooper & O'Quigley, 1982; Raynes & Cooper, 1983). However, we did not find an association between disease extent and baseline CRP concentrations. This is in contrast to the findings of other researchers who have reported such an association in the case of CRP (Cooper & O'Quigley, 1982) and also other acute phase reactant proteins (Harshman *et al.*, 1974; Rosenthal & Sullivan, 1979). The reason for this is uncertain but may relate to the relatively good condition of our extensive disease patients, all of whom were suitable for aggressive induction chemotherapy.

An acute tumour lysis syndrome following chemotherapy has previously been described in a number of different malignancies (Cadman *et al.*, 1977; Cohen *et al.*, 1980; Ettinger *et al.*, 1978) including small cell lung cancer (Vogelzang *et al.*, 1983). This syndrome, characterised by a number of acute biochemical upsets including hyperkalaemia, hypocalcaemia and hyperuricaemia is thought to arise because of chemotherapy induced acute tumour lysis (or necrosis). We hypothesised that the acute tumour necrosis in patients with chemosensitive tumours might induce an acute phase reaction which could be quantified by serial measurement of CRP concentrations. This appears to be the case, with seven out of 10 chemoresponsive patients showing a significant rise in CRP. Interestingly, two of those three chemoresponsive patients who did not show a rise in CRP following chemotherapy had baseline CRP levels within the normal range, perhaps indicating the earlier stage of their tumour. Conversely, in the five chemoresistant patients there was little or no change in CRP concentrations during chemotherapy. Figure 2 illustrates the flat CRP profiles seen in all of the five non-responding patients, presumably a reflection of the reduced or absent necrosis of the resistant tumour.

Furthermore, it should be noted that two of the three patients who died because of acute myelosuppression showed a pronounced rise in CRP concentration with values quadrupling by 72 h. This would also sustain the hypothesis that severe chemotherapy induced tumour necrosis, with associated marrow toxicity results in a marked acute phase reaction at the time of injury and before supervening infection is manifest.

As far as we are aware such a quantifiable acute phase reaction following chemotherapy has not been previously described. One group from Scandinavia (Grutzmeier & Von Schenck, 1986) has reported no change in CRP levels during chemotherapy for acute leukaemia. However, these patients did not have solid tumours, sampling was performed less frequently and may have missed the sharp rise in CRP concentrations we have found, and an unusually high upper limit of CRP was accepted as normal (Milroy *et al.*, 1987).

Although the number of patients in this study is small, there does appear to be evidence of a previously undescribed quantifiable acute phase reaction following chemotherapy in patients with chemoresponsive small cell lung tumours, which is not apparent in resistant patients. If confirmed in larger studies, including other solid tumour types, it might be possible to predict chemoresponse and hence tailor treatment more closely to individual patients needs.

We thank Mrs Elaine Riley for typing this manuscript, Mr R. Carter for statistical advice and the Department of Medical Illustrations, Glasgow Royal Infirmary, for their assistance.

### References

AMOS, R.S., CONSTABLE, T.J., CROCKSON, R.A., CROCKSON, A.P. & McCONKEY, B. (1977). Rheumatoid arthritis: relation of serum C-reactive protein and erythrocyte sedimentation rates to radiographic changes. *Br. Med. J.*, **i**, 195.

BARUAH, B.D. & GOGOI, B.C. (1975). C-reactive protein in malignant tumours. *Ind. J. Cancer*, **12**, 39.

CADMAN, E.C., LUNDBERG, W.B. & BERTINO, J.R. (1977). Hyperphosphataemia and hypocalcaemia accompanying rapid cell lysis in a patient with Burkitt's lymphoma and Burkitt cell leukaemia. *Am. J. Med.*, **62**, 283.

COHEN, L.F., BALOW, J.E., MAGRATH, I.T., POPLACK, D.G. & ZIEGLER, J.L. (1980). Acute tumour lysis syndrome. A review of 37 patients with Burkitt's lymphoma. *Am. J. Med.*, **68**, 486.

COOPER, E.H. & MILFORD WARD, A. (1979). Acute phase reactant proteins as aids to monitoring disease. *Invest. Cell. Pathol.*, **2**, 293.

COOPER, E.H. & STONE, J. (1979). Acute phase reactant proteins in cancer. In *Advances in Cancer Research, Volume 30*, Klein, G. & Weinhouse, S. (eds) p.l. Academic Press: New York.

COOPER, E.H. & O'QUIGLEY, J. (1982). Acute phase reactant protein profiles in cancer: an approach to deciphering their message. In *Marker Proteins in Inflammation*, Allen, R.C., Bienvenu, J., Laurent, P. & Suskind, R.M. (eds) p. 239. Walter de Gruyter & Co: New York.

ETTINGER, D.S., HARKER, W.G., GERRY, H.W., SANDERS, R.C. & SARAL, R. (1978). Hyperphosphataemia, hypocalcaemia and transient renal failure. Results of cytotoxic treatment of acute lymphoblastic leukaemia. *JAMA*, **239**, 2472.

FISCHER, C.L. & GILL, C.W. (1976). Acute phase proteins. In *Serum Protein Abnormalities*, Ritzman, S.E. & Daniels, J.C. (eds) p. 331. Little Brown: Boston.

GRUTZMEIER, S. & VON SCHENCK, H. (1986). C-reactive protein during chemotherapy for acute leukaemia with special reference to non-infective causes of fever. *Med. Oncol. Tumor Pharmacother.*, **3**, 71.

HARSHMAN, S., REYNOLDS, V.H., NEUMASTER, T., PATIKAS, T. & WORRAL, T. (1974). The prognostic significance of serial seromucoid analyses in patients with cancer. *Cancer*, **34**, 291.

MILROY, R., SHAPIRO, D. & SHENKIN, A. (1987). Acute phase reaction following chemotherapy. *Med. Oncol. Tumor Pharmacother.*, **4**, 111 (letter).

POWELL, L.J. (1979). C-reactive protein – a review. *Am. J. Med. Technol.*, **45**, 138.

RAYNES, J.G. & COOPER, E.H. (1983). Comparison of serum amyloid A protein and C-reactive protein concentrations in cancer and non-malignant disease. *J. Clin. Pathol.*, **36**, 798.

ROSENTHAL, C.J. & SULLIVAN, L.M. (1979). Serum amyloid A to monitor cancer dissemination. *Ann. Intern. Med.*, **91**, 383.

VOGELZANG, N.J., NELIMARK, R.A. & NATH, K.A. (1983). Tumour lysis syndrome after induction chemotherapy of small-cell bronchogenic carcinoma. *JAMA*, **249**, 513.

WHICHER, J.T., PERRY, D.E. & HOBBS, J.R. (1978). An evaluation of the Hyland laser nephelometer P.D.Q. system for the measurement of immunoglobulins. *Ann. Clin. Biochem.*, **15**, 77.